# Childhood psychosocial challenges and risk for obesity in U.S. men and women

**DOI:** 10.1038/s41398-018-0341-1

**Published:** 2019-01-17

**Authors:** Melanie M. Wall, Susan M. Mason, Jun Liu, Mark Olfson, Dianne Neumark-Sztainer, Carlos Blanco

**Affiliations:** 1 Department of Psychiatry, Columbia University, 1051 Riverside Drive, New York, NY 10032 USA; 2 New York State Psychiatric Institute, New York, NY USA; 3 Division of Epidemiology, School of Public Health, University of Minnesota, Minneapolis, MN USA; 4 National Institute on Drug Abuse, 6001 Executive Blvd., Rockville, MD 20852 USA

## Abstract

Childhood psychosocial challenges (i.e., adversities, mental and substance use disorders, social challenges) may relate to the onset of obesity and extreme obesity. Identifying the types of psychosocial challenges most strongly associated with obesity could advance etiologic understanding and help target prevention efforts. Using a nationally representative sample of U.S. adults (*N* = 24,350), the present study evaluates relationships between childhood psychosocial challenges and development of obesity and extreme obesity. After mutually controlling, childhood poverty was a risk in men OR = 1.2 (1.0–1.4) and a significantly stronger one in women OR = 1.6 (1.4–1.8); maltreatment increased odds of obesity in both men and women OR = 1.3, 95% CI (1.1–1.4), and specifically increased odds of extreme obesity in women OR = 1.5 (1.3–1.9). Early childrearing (before age 18) was an independent risk factor in both men OR = 1.4 (1.0–1.9) and women OR = 1.3 (1.1–1.5); not finishing high school was the strongest childhood psychosocial challenge risk factor for extreme obesity in both men (OR = 1.6, 1.1–2.2) and women (OR = 2.0, 1.5–2.5). Psychiatric disorders (MDD, anxiety disorder, PTSD) before age 18 were not independently associated with adult obesity in men nor women, but substance use disorders (alcohol or drug) were inversely associated with adult obesity. Individuals who have experienced childhood adversities and social challenges are at increased risk for obesity. Previous findings also indicate that these individuals respond poorly to traditional weight management strategies. It is critical to identify the reasons for these elevated weight problems, and to develop interventions that are appropriately tailored to mitigate the obesity burden faced by this vulnerable population.

## Introduction

In the U.S., nearly 38% of adults have obesity (body mass index, BMI ≥ 30), and nearly 8% have extreme obesity (BMI ≥ 40)^1^. Although childhood obesity, which occurs in approximately 15% of children aged 6 to 11 years, is a well-established risk factor for adult obesity^2–4^, most adults with obesity were not obese or overweight as children. For example, simulations indicate that 40% of non-overweight children aged 13 years will develop obesity by the age of 35^5^.

Much research on childhood risk factors for adult obesity has focused on energy balance factors, including eating and physical activity behaviors, and on reducing exposure to obesogenic environments (e.g., food landscapes, family norms around healthy lifestyle)^6–8^. Less attention has focused on general childhood psychosocial challenges that may increase the risk of obesity in adulthood by heightening vulnerability to obesogenic environments and obesity-promoting behaviors. Adverse childhood experiences, early onset mental health and substance use disorders, and early social challenges, are interrelated childhood psychosocial challenges that may disrupt optimal health development over this critical developmental window. These childhood psychosocial challenges may promote obesity through several pathways. For example, several studies have suggested that childhood adversities may lead to obesity via affect dysregulation (diminished capacity to cope with distress), which leads to obesity-promoting coping strategies such as ingestion of highly palatable foods that trigger dopaminergic reward responses^9–12^. Other plausible mechanisms include physiologic impacts of these challenges on the hypothalamic–pituitary–adrenal axis, and social mechanisms such as truncation of socioeconomic attainment.

**Fig. 1:**
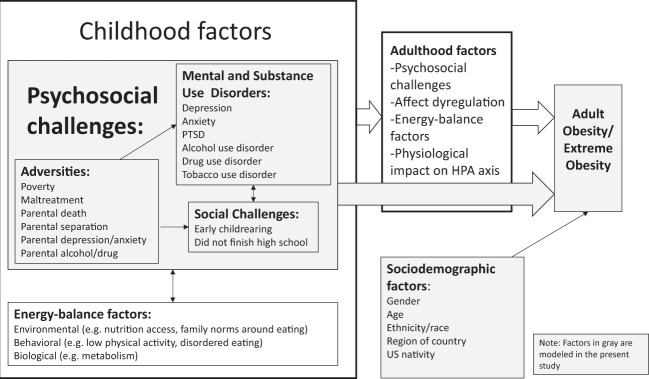
Conceptual Model for childhood psychosocial challenges predicting adult obesity and extreme obesity

Adverse childhood experiences include external events that directly threaten the body or the social emotional safety of the child or family. We include poverty in this list of adverse experiences as has been recently advocated^13^. Growing evidence indicates that adverse childhood experiences, such as maltreatment^14–16^, parental death or separation^17–19^, and poverty^20,21^, are important risk factors for later obesity. Childhood mental and substance use disorders are another common set of challenges that have been linked to obesity. These include early onset major depressive disorder (MDD)^22–24^ and tobacco use disorder^25–27^. Little prior research has examined the association of these mental health and substance use disorders to obesity within the context of other correlated problems such as adverse childhood experiences. Finally, other childhood social challenges, also correlated with adverse childhood experiences and mental health and substance use disorders, such as early childrearing^28^ and not finishing high school^29,30^ have been found in some studies to predict obesity. What remains unclear is whether these challenges cumulatively contribute to obesity risk, or if certain individual challenges play an outsized role. Specifically, prior research has not evaluated the independent effects of these challenges by accounting for their correlations with each other. Further, no studies have used a U.S. nationally representative sample to evaluate their impact in the general population. Figure 1 illustrates how these childhood psychosocial challenges may lead to the development of adult obesity.

Certain psychosocial challenges disproportionately burden women^31^, and some may differentially effect the risk of obesity in women vs. men^32–34^. Thus, comparisons of challenges across genders —both their prevalence and associations with weight status—could help explain the higher prevalence of extreme obesity in women vs. men; as compared to men, women in the U.S. experience an 80% higher prevalence of extreme obesity (9.9% vs. 5.5%)^35^. However, the role of psychosocial childhood challenges in the gender disparity of adult obesity has not been previously examined.

Using a large nationally representative sample, the goal of this study was to evaluate the associations of a wide class of psychosocial challenges experienced during childhood including childhood adversities, early mental health and substance use disorders, and social challenges with development of obesity and extreme obesity in men and women. We focus on adults who did not experience being overweight as children to isolate associations with onset of obesity. While mediators of these childhood psychosocial challenges exist in adulthood (Fig. 1), we do not try to control for them (e.g., adult environment, psychopathology, or behaviors) as our intention is to capture the total effect of these childhood psychosocial challenges on adult obesity combined across all their mechanisms of action. Results will advance the understanding of potential social and psychological origins of obesity, and identify groups for targeted prevention efforts.

## Materials and methods

### Sample

The National Epidemiological Survey on Alcohol and Related Conditions III (NESARC-III) is a nationally representative in-person interview study of 36,309 adults of age 18 years and older residing in households and selected group quarters. Data collection occurred in 2012–2013. As detailed elsewhere^36^, probability sampling was used to select respondents. Screener- and person-level response rates were 72.0% and 84.0%, respectively, yielding a total response rate of 60.1% (*N* = 36,309). Non-response was adjusted by weighting as detailed elsewhere^37^. The study protocol was approved by National Institutes of Health and Westat Institutional Review Boards.

Of 36,309 in the base sample, we exclude adults over the age of 65 (*n* = 5,714) and women who were currently pregnant or within 1 year postpartum (*n* = 1,302), as obesity may be etiologically distinct during these life stages, and those missing BMI (the primary outcome; *n* = 563), leaving an analytic sample of *N* = 28,730 (13,393 men and 15,337 women). Primary regression models further restricted to those who reported that they were not overweight prior to age 13 years (*N* = 24,350, 11,481 men and 12,869 women), to ensure the correct temporal order from challenges to obesity onset.

### Measures

#### Adult current weight status

The primary outcomes for this study were extreme obesity (BMI ≥ 40) and obesity (BMI ≥ 30)^38^. Descriptive analyses also included non-overweight (BMI ≥ 18.5 and BMI < 30), and underweight (BMI < 18.5) categories. BMI was derived from self-reported height and weight and calculated as weight in kilograms divided by the square of height in meters. Self-reported height and weight have been found to be highly correlated with measured height and weight, suggesting that self-report is a valid source of this information in large epidemiologic studies^39^.

#### Childhood overweight status

Directly after being asked to report their current height and weight, adult respondents were asked “When you were growing up, that is, before you were 13 years old, were you overweight?” Response options were yes or no.

#### Socio-demographic characteristics

Analyses were adjusted for socio-demographic characteristics that may be common causes of childhood challenges and obesity. Age was categorized as 18–34, 35–49, and 50–64 years; census region of current residence as North, Midwest, South, West; race/ethnicity as Hispanic (any race), and Non-Hispanic white, black, American Indian/Alaska Native, Asian/Native Hawaiian/Other Pacific Islander; Nativity as U.S. born vs. not.

#### Childhood psychosocial challenges

Childhood challenges (before age 18) included: six adverse childhood experiences (poverty, maltreatment, parental death, parental separation, parental mood/anxiety disorder, parental substance use disorder), six early onset mental health and substance use disorders (depression, anxiety, post-traumatic stress disorder (PTSD), alcohol, drug, and tobacco use disorder), and two social challenges (early childrearing and not finishing high school). Below we describe the measurement of each.

*Poverty* during childhood was considered present when respondents answered yes to the following: “Before you were 18 years old, was there ever a time when your family received money from government assistance programs like welfare, food stamps, general assistance, Aid to Families with Dependent Children, or Temporary Assistance for Needy Families?”

*Childhood maltreatment* by a parent or caregiver was measured by questions adapted from widely used, well-validated scales^40^ assessing physical, verbal, and sexual abuse. Participants were asked the frequency (never, almost never, sometimes, fairly often, and very often) with which they had the maltreatment experiences before age 18 and we used established frequency cut-points^41,42^, to define the presence or absence of each type of abuse. An overall “any childhood maltreatment” variable was created and considered positive if any of the three types of abuse were positive. We also created a combined maltreatment variable that assessed the maximum frequency of any of the three types of abuse.

*Parental death* was indicated when a respondent answered yes to the following question “Did either of your (biological/adoptive) parents die before you were 18?”.

*Parental separation* was assessed by asking participants which (biological/adoptive) parents they lived with, and whether their parents were ever divorced or separated before the participant turned 18. The variable coded as: (1) “Never together”, respondents indicating they never lived with both biological or both adoptive parents or (2) “Divorced/separated”, respondents indicating parental divorce/separation, and (3) “Always together”, respondents indicating they lived with both parents and those parents never divorced or separated.

*Parental mood or anxiety disorder* was assessed referring to biological parents and whether either father or mother ever had times when either was depressed, had behavior problems, or had anxious problems.

*Parental substance use disorder* was assessed referring to either father or mother and whether they had ever been an alcoholic or ever had drug problems.

*Onset of mental health and substance use disorders before 18:* Major depressive disorder (MDD), anxiety disorder (including specific phobia, general anxiety disorder, and social anxiety disorder), post traumatic stress disorder (PTSD), alcohol use disorder, drug use disorder, and tobacco use disorder were assessed by DSM-5 criteria (APA, 2013) using the Alcohol Use Disorder and Associated Disabilities Interview Schedule-5 (AUDADIS-5), a computer-assisted interview that assesses mental health and substance use disorders with good reliability and validity^43^. Once lifetime presence of a disorder was indicated, onset age before 18 was determined.

*Early childrearing* for both males and females was considered present when respondents answered age 17 or younger to the following: “How old were you when your first child was born or when your first step, adopted, or foster child began to live with you (Report earliest age if experienced more than one of these events)”.

*Not finishing high school* was coded as positive when respondents indicated that their highest educational attainment was less than high school.

### Statistical analysis

Descriptive analyses in the full analytic sample included estimation of the distribution of adult obesity and extreme obesity by childhood overweight status, age, region, race/ethnicity, and U.S. birthplace, overall and stratified by sex. Differences by gender were tested with *χ*^2^ tests and associations were tested with logistic regression mutually controlling for all demographics.

Analyses of associations between childhood psychosocial challenges and onset of obesity in adulthood were restricted to adults who were not overweight before the age of 13 (*N* = 24,350) to reduce potential confounding by childhood overweight status. Logistic regression was used to model the association of each individual stressor with obesity and extreme obesity in separate models, stratified by gender, controlling for age, region, race/ethnicity, and U.S. birthplace. Tetrachoric correlations between the childhood challenges were calculated and examined for multicollinearity that would motivate combining of certain groups of challenges before performing multivariate analysis. To identify the childhood psychosocial challenges most strongly associated with adult obesity, logistic regression models were used, mutually adjusting for all childhood challenges, or in some cases groups of challenges. Differential associations by gender were tested with gender-by-predictor interaction terms. Finally, to explore childhood psychosocial challenges as the reason for gender differences in extreme obesity, we estimated the natural indirect effect on the risk difference scale^43-46^, and used it to estimate the proportion of the total absolute difference in extreme obesity risk between men and women attributable to childhood psychosocial challenges.

Data analysis was conducted from December 2017 to September 2018. All prevalence estimates, standard errors, odds ratios, and 95% CIs were estimated using SURVEY procedures in SAS/STAT software, Version 9.4, to account for sampling weights and the clustered design of the survey.

## Results

### Obesity and extreme obesity

**Fig. 2:**
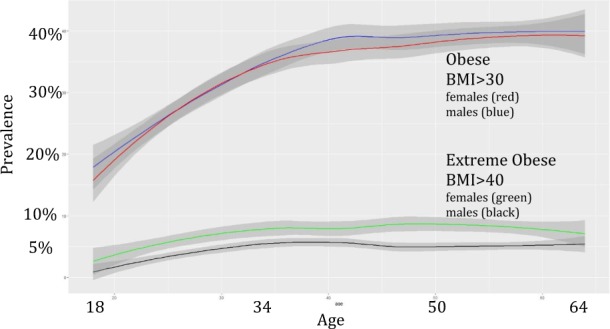
Prevalence with 95% confidence intervals of obesity and extreme obesity by age and gender in the U.S. population (2012–2013)

In this representative sample of U.S. adults aged 18–64, an estimated 34.2% had obesity and 5.9% had extreme obesity (Table 1). In both men and women, the prevalence of both obesity and extreme obesity increased in adulthood until age 35 then leveled off (Fig. 2, Supplemental Table 1). The prevalence of obesity was similar in men and women, but extreme obesity was significantly more common in women (7.4%) than men (4.6%) across all ages (Fig. 2). Being overweight as a child was associated with high prevalence of adult obesity (59.3%) and extreme obesity (16.6%), but a large majority of adults who had obesity (73.2%, 95% CI 72.6–73.6) were not overweight as children.

**Table 1:** Prevalence of adult (age 18–64) weight status and recalled childhood weight status in U.S. population in 2012–2013 overall and by gender (*N* = 28,730)

U.S. sample age 18–64	Total (*n* = 28,730)		Men (*n* = 13,393)		Women^a^ (*n* = 15,337)		Test of gender difference
	*N*	% (se)	*N*	% (se)	*N*	% (se)	
Adult current weight status^b^							
Underweight (BMI < 18.5)	286	1.05 (0.08)	71	0.61 (0.08)	215	1.52 (0.14)	<0.0001
Normal weight (BMI 18.5–30)	18193	64.69 (0.54)	8741	64.98 (0.65)	9452	64.38 (0.67)	
Obese (not extreme) (BMI 30–40)	8343	28.33 (0.42)	3960	29.83 (0.57)	4383	26.76 (0.51)	
Extreme Obese (BMI > 40)	1908	5.93 (0.23)	621	4.57 (0.23)	1287	7.35 (0.34)	
Childhood overweight status (age < 13)^c^							
Overweight	4325	15.48 (0.29)	1885	14.66 (0.41)	2440	16.34 (0.42)	0.0049
Not overweight	24350	84.52 (0.29)	11481	85.34 (0.41)	12869	83.66 (0.42)	
Adult weight status among those overweight in childhood							
	Total (*n* = 4325)		Men (*n* = 1885)		Women (*n* = 2440)		
Obese (>30)	2597	59.28 (1.12)	1111	59.97 (1.46)	1486	58.64 (1.54)	0.45
Extreme Obese (>40)	767	16.64 (0.77)	261	13.93 (0.99)	506	19.19 (1.11)	0.0003
Adult weight status among those NOT overweight in childhood							
	Total (*n* = 24,350)		Men (*n* = 11,481)		Women (*n* = 12,869)		
Obese (>30)	7622	29.62 (0.52)	3457	29.98 (0.63)	4165	29.24 (0.66)	0.35
Extreme obese (>40)	1138	3.97 (0.19)	358	2.96 (0.21)	780	5.04 (0.28)	<0.0001

^a^Women who were currently or recently pregnant within past year (*n* = 1302) were not included

^b^Body mass index (BMI) derived from self-reported height and weight in response to question “Please tell me your height and weight in pounds as these are important factors for this survey.” *N* = 652 subjects were not included due to missing height or weight and 11 subjects were not included due to outlying values: BMI <12 or height <3 feet. Lowest underweight BMI in sample = 13, highest severe obese BMI in sample = 85

^c^After being asked to report their current height and weight, adult respondents were asked “When you were growing up, that is, before you were 13 years old, were you overweight? Yes/No”

### Race/ethnicity, region, and U.S. nativity

Subsequent results are estimated in adults who were not overweight as children. We found an increased odds of onset of obesity after childhood among black, Native American, and Hispanic women and men relative to non-Hispanic white participants of the same gender. Asian race/ethnicity and foreign birth was associated with reduced obesity risk. South and Midwest residence was associated with obesity after controlling for other demographic characteristics in men but not in women (Table 2). Disparities in extreme obesity associated with black (vs. non-Hispanic white) race and US (vs. foreign) birth were greater for women than men. Similar demographic associations with adult obesity and extreme obesity were also found in the full sample including those who experienced being overweight as children (Supplemental Table 1).

**Table 2:** Distribution of demographics and childhood psychosocial challenges by adult obesity among U.S. adults age 18–64 who were NOT overweight in childhood before age 13 (*N* = 24,350)

		Men					Women				
		Obese (*N* = 3457)		Not obese (*N* = 8024)			Obese (*N* = 4165)		Not obese (*N* = 8704)		
		*n*	%	*n*	%	OR^a^ (95% CI)	*n*	%	*n*	%	OR^a^ (95% CI)
Demographic characteristics											
Age	18–34	908	24.8	3358	40.8	1	1038	21.6	3341	36.1	1
	35–49	1287	36.2	2394	30.0	**2.12 (1.86, 2.41)**	1596	37.8	2879	32.4	**2.18 (1.89, 2.51)**
	50–64	1262	38.8	2272	29.1	**2.34 (2.07, 2.71)**	1531	40.4	2484	31.3	**2.46 (2.14, 2.82)**
Region	North	441	16.0	1152	18.5	1	520	16.4	1281	18.5	1
	Midwest	722	23.1	1634	20.7	**1.26 (1.06, 1.50)**	825	21.3	1712	20.5	1.12 (0.92, 1.36)
	South	1485	38.9	3087	36.0	**1.17 (1.00, 1.37)**	1959	41.8	3356	35.4	1.15 (0.95, 1.40)
	West	809	21.9	2151	24.6	1.01 (0.80, 1.29)	861	20.3	2355	25.4	0.94 (0.76, 1.17)
Race/ethnicity	White	1640	62.5	4095	63.6	1	1637	56.2	4501	64.3	1
	Black	859	13.9	1641	11.3	**1.35 (1.18, 1.54)**	1497	21.4	1619	9.9	**2.68 (2.35, 3.06)**
	Amer. Indian	53	1.7	102	1.1	**1.55 (1.09, 2.23)**	83	2.6	117	1.6	**2.03 (1.43, 2.88)**
	Asian	83	2.8	570	7.8	**0.53 (0.36, 0.77)**	59	2.2	636	9.0	**0.43 (0.31, 0.61)**
	Hispanic	822	19.0	1616	15.9	**1.78 (1.51, 2.10)**	889	17.6	1831	15.1	**1.97 (1.70, 2.29)**
Birthplace	U.S.	2862	84.9	6287	80.1	1	3555	86.6	6816	80.1	1
	Other	594	15.0	1733	19.8	**0.63 (0.54, 0.73)**	609	13.4	1888	19.8	**0.59 (0.50, 0.70)**
Childhood psychosocial challenges before 18 years old											
Poverty	Yes	703	18.8	1456	16.2	**1.18 (1.03, 1.35)**	1219	25.1	1603	14.9	**1.78 (1.59, 1.99)**
	No	2673	81.1	6393	83.7	1	2839	74.8	6948	85.0	1
Maltreatment (verbal, physical, or sexual abuse)	Yes	1010	29.0	2003	23.6	**1.25 (1.12, 1.40)**	1503	37.8	2523	28.5	**1.37 (1.23, 1.51)**
	No	2421	70.9	5961	76.3	1	2608	62.1	6102	71.5	1
Parental death	Yes	321	8.7	712	8.0	1.06 (0.88, 1.26)	452	9.5	700	7.6	1.13 (0.96, 1.35)
	No	3085	91.2	7189	91.9	1	3630	90.4	7858	92.3	1
Parental separation	Never together	561	12.2	1297	12.8	0.95 (0.82, 1.10)	914	16.7	1329	12.1	**1.31 (1.05, 1.33)**
	Divorced	707	20.2	1753	21.4	0.96 (0.82, 1.11)	991	23.6	1973	21.3	**1.18 (1.05, 1.33)**
	Always together	2188	67.4	4964	65.7	1	2257	59.5	5398	66.5	1
Parental mood/anxiety disorder	Yes	1339	41.3	3093	40.1	1.06 (0.95, 1.18)	1905	49.3	3904	47.0	**1.15 (1.04, 1.28)**
	No	2118	58.7	4931	59.9	1	2260	50.7	4800	53.0	1
Parental substance use disorder	Yes	1151	32.9	2533	31.0	1.04 (0.93, 1.16)	1624	39.2	3044	34.9	**1.16 (1.03, 1.30)**
	No	2306	67.1	5491	69.0	1	2541	60.8	5660	65.1	1
Major depressive disorder^b^	Yes	86	2.4	294	3.8	0.77 (0.56, 1.07)	239	6.2	547	6.7	**1.28 (1.00, 1.64)**
	No	3371	97.5	7730	96.2	1	3926	93.7	8157	93.2	1
Anxiety disorder^b^	Yes	171	5.1	433	5.7	0.96 (0.77, 1.20)	394	10.4	815	10.1	1.13 (0.96, 1.32)
	No	3286	94.9	7591	94.3	1	3771	89.6	7889	89.9	1
PTSD^b^	Yes	53	1.5	99	1.1	1.23 (0.79, 1.94)	181	4.2	262	3.0	**1.56 (1.27, 1.93)**
	No	3404	98.5	7925	98.9	1	3984	95.8	8442	97.0	1
Alcohol use disorder^b^	Yes	111	3.5	301	4.1	0.91 (0.71, 1.17)	78	2.2	261	3.2	0.80 (0.60, 1.06)
	No	3346	96.5	7723	95.9	1	4087	97.8	8443	96.8	1
Drug use disorder^b^	Yes	85	2.8	283	3.8	0.79 (0.61, 1.00)	66	1.8	185	2.2	0.92 (0.62, 1.37)
	No	3372	97.2	7741	96.2	1	4099	98.2	8519	97.8	1
Tobacco use disorder^b^	Yes	140	4.8	344	4.9	1.02 (0.77, 1.35)	136	3.8	293	3.5	1.20 (0.95, 1.51)
	No	3317	95.1	7680	95.1	1	4029	96.1	8411	96.4	1
Childrearing	Yes	136	2.9	213	1.8	**1.40 (1.03, 1.91)**	680	12.9	799	6.75	**1.61 (1.38, 1.86)**
	No	3317	97.0	7785	98.1	1	3480	87.0	7892	93.2	1
Did not finish High School	Yes	566	14.4	1150	12.3	**1.16 (1.01, 1.32)**	725	15.3	1031	10.0	**1.68 (1.47, 1.92)**
	No	28sample of U.S. adults91	85.5	6874	87.7	1	3440	84.7	7673	90.0	1

^a^ORs are adjusted for demographic variables: age, region, race/ethnicity, and birthplace

^b^Onset of disorder occurring at age <18

Bold indicates significance at p<.05

### Childhood psychosocial challenges and obesity

The prevalence of all childhood psychosocial challenges, except substance use disorder, was higher in women with obesity as compared to men with obesity (maltreatment 37.8% for women vs. 29.0% for men; childhood poverty 25.1% for women vs. 18.8% for men; parental separation 40.5% for women vs. 32.6% for men; parental mood/anxiety problems 49.3% for women vs. 41.3% for men; parental alcohol/drug problems 39.2% women vs. 32.9% for men; MDD onset before age 18 6.2% for women vs. 2.4% for men; anxiety disorder 10.4% for women vs. 5.1% for men; PTSD 4.2% for women vs. 1.5% for men; early childrearing 12.9% for women vs. 2.9% for men; and not finishing high school 15.3% for women vs. 14.4% for men, Table 2). Similar differences in childhood challenges were found by gender in those who did not have adult obesity and in the full sample (Supplemental Table 2), with the exception that in the full sample men were more likely than women to not finish high school.

In those not overweight as children, childhood poverty, maltreatment, early childrearing, and not finishing high school were all associated with increased odds of adult obesity in both men and women, after controlling for socio-demographic characteristics. Parental separation, parental mood/anxiety problems, parental alcohol/drug problems, MDD before age 18, and PTSD before age 18 were associated with obesity only in women (Table 2). Parental death, anxiety disorder, and tobacco use disorder before 18 were not associated with obesity after controlling for demographics. Onset before age 18 of alcohol use disorder in women, and drug use disorder in men trended toward being protective for obesity indicating decreased likelihood of adult obesity though not reaching statistical significance.

Supplemental Table 3 presents the correlations between the 14 different childhood psychosocial challenges. Associations between factors were similar for men and women. The correlation of early onset of internalizing disorders: MDD, anxiety disorders, and PTSD were all >0.40 motivating their combination for subsequent analysis; similarly, the correlation between early onset of alcohol and drug use disorders was >0.60 and so they were combined.

After mutually controlling for all childhood psychosocial challenges as well as socio-demographic characteristics (Table 3), childhood poverty remained associated with onset of obesity in men (OR = 1.16; 95% CI: 1.00, 1.35) and even more strongly in women (OR = 1.57; 95% CI: 1.39, 1.77; *p*-value gender interaction <0.001). Childhood maltreatment was associated similarly with obesity in both men (OR = 1.26; 95% CI: 1.11–1.43) and women (OR = 1.25; 95% CI: 1.11–1.40). In women, the increased odds of obesity were similar regardless of the type of abuse; in men, verbal and physical abuse, but not sexual abuse, were associated with obesity (Supplemental Table 4). Early childrearing was an independent risk factor for obesity in men (OR = 1.37; 95% CI: 1.00–1.87) and women (OR = 1.29; 95% CI: 1.10–1.53). In women, not finishing high school (OR = 1.48; 95% CI: 1.30, 1.70) was an independent risk factor for obesity, but not for men. After mutual adjustment for all psychosocial challenges, combined MDD, anxiety disorder, or PTSD before age 18 was not associated with obesity in women nor men, but combined alcohol or drug use disorder was protective in both men (OR = 0.80; 95% CI:0.64–1.00) and women (OR = 0.68 (0.53,0.86). In mutually adjusted models, parental death, parental separation/divorce, parental psychopathology, and tobacco use disorder before 18 were not associated with obesity in men or women.

**Table 3:** Mutually adjusted demographic and childhood psychosocial challenges predicting Obesity and Extreme Obesity by gender among U.S. adults age 18–64 who were NOT overweight in childhood (*N* = 24,350)

		Obese vs. not obese		Difference in OR by gender	Extreme obese vs. not extreme obese		Difference in OR by gender
		Male	Female	*p*-Value all interaction	Male	Female	*p*-Value all interaction
		OR^a^ (95% CI)	OR^a^ (95% CI)		OR^a^ (95% CI)	OR^a^ (95% CI)	
Demographics							
Age	35–49	**2.07 (1.81, 2.36)**	**2.23 (1.95, 2.56)**	0.84	**1.90 (1.32, 2.73)**	**1.87 (1.50, 2.33)**	0.77
(refs. ^18–34^)	50–64	**2.30 (1.99, 2.65)**	**2.60 (2.25, 3.00)**	0.33	**2.21 (1.55, 3.15)**	**1.95 (1.54, 2.46)**	0.49
Region (ref = North)	Midwest	**1.27 (1.07, 1.51)**	1.11 (0.91, 1.35)	0.29	1.17 (0.77, 1.76)	1.03 (0.70, 1.50)	0.61
	South	**1.18** (**1.00, 1.38)**	1.13 (0.93, 1.37)	0.64	1.06 (0.7, 1.62)	1.11 (0.80, 1.53)	0.52
	West	1.02 (0.80, 1.29)	0.93 (0.75, 1.15)	0.75	1.06 (0.63, 1.81)	0.95 (0.63, 1.43)	0.75
Race/ethnicity (ref = White)	Black	**1.28 (1.11, 1.48)**	**2.31 (2.02, 2.64)**	**0.00**	1.23 (0.87, 1.73)	**2.24 (1.73, 2.90)**	**0.02**
	Amer. Indian	**1.49 (1.05, 2.10)**	**1.80 (1.23, 2.62)**	0.78	1.75 (0.83, 3.7)	**2.18** (**1.32, 3.59)**	0.63
	Asian	**0.53** (**0.37, 0.76)**	**0.44** (**0.31, 0.62)**	0.12	0.59 (0.21, 1.67)	**0.26 (0.09, 0.77)**	0.23
	Hispanic	**1.70 (1.44, 2.02)**	**1.73 (1.48, 2.02)**	0.28	1.35 (0.86, 2.11)	**1.38 (1.00, 1.89)**	0.96
Birthplace (ref = U.S.)	Other	**0.63 (0.55, 0.73)**	**0.65 (0.54, 0.77)**	0.86	**0.29 (0.18, 0.47)**	**0.56 (0.39, 0.80)**	**0.05**
Childhood psychosocial challenges before 18 years old							
Poverty	Yes	**1.16 (1.00, 1.35)**	**1.57 (1.39, 1.77)**	**0.00**	1.33 (0.94, 1.88)	**1.64 (1.27, 2.11)**	0.33
Maltreatment (verbal, physical, or sexual abuse)	Yes	**1.26 (1.11, 1.43)**	**1.25 (1.11, 1.40)**	0.91	0.97 (0.73, 1.3)	**1.46 (1.18, 1.79)**	**0.02**
Parental death	Yes	1.05 (0.88, 1.26)	1.00 (0.84, 1.20)	0.73	0.99 (0.66, 1.5)	1.02 (0.75, 1.39)	0.91
Parental separation (ref = “Always together”)	Never together	0.88 (0.75, 1.02)	1.06 (0.91, 1.24)	0.23	0.82 (0.52, 1.3)	0.90 (0.70, 1.15)	0.81
	Divorced	0.91 (0.78, 1.05)	1.02 (0.90, 1.15)	0.86	0.85 (0.58, 1.24)	0.90 (0.72, 1.14)	0.94
Parental mood/anxiety disorder	Yes	1.02 (0.91, 1.15)	1.05 (0.94, 1.17)	0.75	1.05 (0.82, 1.36)	1.07 (0.88, 1.32)	0.91
Parental substance use disorder	Yes	1.00 (0.89, 1.12)	1.00 (0.88, 1.13)	0.97	1.25 (0.88, 1.77)	1.06 (0.84, 1.33)	0.43
MDD/Anxiety/PTSD^b^	Yes	0.93 (0.78, 1.11)	1.09 (0.94, 1.26)	0.21	1.33 (0.90, 1.97)	1.26 (0.96, 1.66)	0.82
AUD/DUD^c^	Yes	**0.80 (0.64, 0.99)**	**0.68 (0.53, 0.86)**	0.33	0.87(0.52,1.44)	0.62 (0.36, 1.06)	0.33
Tobacco use disorder	Yes	1.01 (0.76, 1.35)	1.04 (0.82, 1.30)	0.90	1.30 (0.77, 2.21)	0.91 (0.55, 1.5)	0.34
Childrearing	Yes	**1.37 (1.00, 1.87)**	**1.29 (1.10, 1.53)**	0.76	1.92 (0.99, 3.71)	1.11 **(**0.87, 1.4)	0.12
Did not finish High School	Yes	1.14 (0.99, 1.3)	**1.48 (1.30, 1.70)**	**0.01**	**1.59 (1.15, 2.21)**	**1.95 (1.52, 2.49)**	0.32
C-INDEX		0.63	0.68		0.64	0.70	

^a^ORs are mutually adjusted for ALL demographic and childhood psychosocial challenges in the tables, listwise deletion *N* = 10,920 for men, *N* = 12,184 for women

^b^Indicator of whether any of major depressive disorder (MDD), anxiety disorder, or PTSD onset before age 18

^c^Indicator of whether either alcohol use disorder (AUD) or drug use disorder (DUD) onset before age 18

### Childhood psychosocial challenges and extreme obesity

Not finishing high school was the only childhood stressor associated with extreme obesity in men (OR = 1.59; 95% CI: 1.15–2.21) after mutually adjusting for all other childhood psychosocial challenges and socio-demographic characteristics (Table 3). For women, the independent childhood risk factors for extreme obesity were not finishing high school (OR = 1.95; 95% CI: 1.52–2.49), poverty (OR = 1.64; 95% CI; 1.27, 2.11), and maltreatment (OR = 1.46; 95% CI: 1.18–1.79). Using a multiple mediator model, which takes account of differential strength of effects by gender as well as different prevalence of childhood psychosocial challenges by gender, we estimated that 26% of the gender difference in extreme obesity was explained by the childhood psychosocial challenges. Specifically, the total gender effect in extreme obesity was a Risk Difference (RD) of 2.08% (5.04% in women minus 2.96% in men); after all the mediating effects of the childhood psychosocial challenges were taken into account, the remaining “direct” effect of gender on extreme obesity (i.e., the effect not operating through childhood psychosocial challenges) was estimated to be an RD of 1.54%, or 74% of the total 2.08%, leaving 26% explained by childhood psychosocial challenges.

## Discussion

In a large and nationally representative sample, multiple childhood psychosocial challenges were independently associated with onset of obesity and extreme obesity in adulthood, particularly in women. Importantly, by designing the analyses to examine these associations in U.S. adults who were not overweight as children we are able to attribute a causal ordering such that the stressors occur before the onset of obesity. The fact that most (73.2%) of adults with obesity were not overweight in childhood highlights the importance of this developmental window for obesity prevention. The well-documented challenges of weight loss and obesity treatment further support the urgent need for a prevention focus during childhood and adolescence. Our findings are consistent with a growing body of research suggesting that childhood experiences may have impacts across the life course. Identifying specific childhood psychosocial challenges that are salient for obesity can help target prevention programs to those at highest risk for obesity, before significant weight gain has occurred. Our results add to the existing literature on risk factors for obesity in several ways. First, we examined multiple psychosocial challenges simultaneously. Second, we estimated these links in a nationally representative sample. Third, we included an investigation of extreme obesity. Finally, we compared these associations between men and women, and estimated the contribution of these challenges to gender disparities in extreme obesity.

Much prior work examining childhood psychosocial challenges has focused on childhood maltreatment. Two recent meta analyses have found that, across >50 studies, childhood maltreatment was associated with increased odds of adult obesity^14,47^. However, most prior work has not accounted for other childhood challenges, or childhood overweight status, leaving unanswered the question of whether the childhood maltreatment association is due primarily to confounding by early body weight or other correlated psychosocial factors. By mutually examining multiple psychosocial challenges in a sample that was not overweight as children, we were able to address some of these concerns and identify an independent effect not mediated through childhood mental health disorders or other social challenges. Further, only one of the studies included in the meta-analyses was nationally representative^48^. Finally, our study demonstrated important gender differences in links between childhood psychosocial challenges and later obesity, consistent with some^34^ but not all^16^ prior studies. These psychosocial challenges may help to explain gender disparities in extreme obesity. Examining risk factors of extreme obesity is an important contribution, given the high health risks faced by those with extreme obesity^49–51^.

One motivation for work on the links from mental health and substance use disorders to obesity has been an effort to understand pathways from childhood maltreatment to obesity risk^52^. There is some limited evidence of an association between lifetime mood disorders and obesity^53^, but little evidence for substance use disorder associations with obesity^53,54^. In the current study, in models only adjusting for demographics, childhood MDD and PTSD were risk factors for obesity in women, but this relationship did not remain significant after controlling for other childhood psychosocial challenges. These findings suggest that childhood challenges that influence psychiatric and substance use disorders may have the greater salience for obesity. Of note, substance use disorders (alcohol and drug) before 18 were found to be protective for obesity after controlling for other childhood challenges. This finding is consistent in men with prior findings that men with drug use disorders are less likely to become overweight or obese^55^. It is possible that alcohol or drug use may be an alternate coping mechanism to overeating, which, while also maladaptive, does not confer the same risk of obesity.

To the best of our knowledge, this is the first study to examine the association of early childrearing with obesity in a nationally representative sample, and the first to consider this association among men. We found that both men and women who had a child before age 18 had increased odds of developing obesity as adults. Although we cannot know with certainty, we assume that most women reporting early childrearing in our sample were rearing their own biological children, and thus also experienced an early pregnancy. Prior evidence shows that early adversities are associated with sexual risk-taking and younger age at pregnancy^56,57^. Early pregnancy may be one pathway by which other childhood psychosocial challenges influence later obesity risk. Further, recent evidence finds that women with early adversities are more likely to gain excessive weight during pregnancy^58,59^, indicating that these psychosocial challenges may operate synergistically to elevate obesity risk. Somewhat unexpectedly, the effect estimate for men was similarly strong. This suggests that mechanisms unrelated to pregnancy may also contribute to obesity risk, perhaps related to adverse effects of time constraints and parenting challenges on eating and physical activity.

Our finding that both childhood poverty and not finishing high school were stronger risk factors for obesity in U.S. women than men is consistent with a recent meta-analysis of 14 (mostly non-U.S.) studies that also concluded there are lasting childhood effects of poverty on obesity in women but not men^60^. In the present study, we further stratified population groups, finding substantially higher rates of obesity and extreme obesity in black compared to white adults and in black women compared to black men. This pattern of results suggests that race and childhood psychosocial challenges may independently contribute to obesity in black women. Given the contribution of obesity to the leading causes of death, these findings are relevant to the wider literature on socioeconomic disparities in mortality risk^61^.

The present study has some limitations. First, our study relies on self-reported height and weight (BMI) which may have systematic downward bias^62^. However, data collection was done by face-to-face interviews, possibly minimizing risk of gross misreporting of height and weight, and previous analyses have found similar rates of obesity based on self-report and measured weight^35^. Second, retrospective recall of childhood psychosocial challenges and childhood weight status may be subject to recall bias. Nevertheless, recall of these types of childhood experiences is the standard approach in most large studies, as other types of measures (prospective in childhood or confirmation via objective means such as child welfare reports) are infeasible, ethically problematic, and lead to under-ascertainment^63,64^. Third, questions around energy-balance factors (Fig. 1) during childhood were not asked on the survey and so could not be investigated as potential mediators. Finally, although we established a temporal order from childhood challenges to obesity with some confidence, the time order of different childhood challenges relative to each other could not be unpacked, nor could adult factors be incorporated as their time order was unknown with respect to the onset of obesity. Future research in longitudinal data should consider how early childhood psychosocial challenges (e.g., childhood maltreatment) operate through later ones (e.g., not finishing high school) to influence obesity risk. Future research should also identify which pathways leading to adult risk factors are strongest to better specify mechanisms by which the childhood psychosocial challenges exert their influence on adult obesity.

Multiple biological, psychological, and social mechanisms may operate together to link childhood psychosocial challenges to adult obesity. For example, childhood maltreatment may lead to obesity via both direct physiologic impacts of HPA-axis regulation, through disruption of normal development of executive function, which in turn may result in truncated educational attainment, or through increased sexual risk taking which can result in early childrearing. Our findings suggest a possible etiologic role of childhood exposure to maltreatment in both males and females that is not explained or mediated by other related psychosocial challenges, and appears to have a particularly strong role for extreme obesity in women. Our findings also highlight the high risk for obesity among those experiencing childhood poverty, low educational attainment, and early childrearing. Populations with these risk patterns should be considered in obesity prevention and treatment strategies. At the same time, traditional weight management interventions may be less effective for these populations^65–67^. Efforts are needed both to reduce the prevalence of these psychosocial challenges and to tailor weight management interventions to the specific needs of individuals who have experienced them. In addition to general school-based programs that involve promotion of physical activity and healthy dietary habits^68^, there may be a role for more intensive primary care based preventative interventions targeting normal weight children and adolescents with multiple known psychosocial risk factors for adult obesity. This tailoring will require greater understanding of the unique mechanisms linking psychosocial challenges to obesity and an emphasis on identifying potential intervention targets to inform optimal prevention and treatment approaches that appropriately address the needs of these vulnerable populations. Future work should investigate the mechanisms linking these psychosocial risk factors to obesity and test the effectiveness of obesity interventions tailored specifically to address these mechanisms.

## Supplementary information

Supplemental Tables
