# Supplementary material for: Childhood psychosocial challenges and risk for obesity in U.S. men and women
Source: Transl Psychiatry. 2019 Jan 17;9:16. doi: 10.1038/s41398-018-0341-1 (PMC6336849; doi:10.1038/s41398-018-0341-1)
Supplement: Supplementary file 1 — Supplemental Tables [file 41398_2018_341_MOESM1_ESM.docx]

Supplemental Table 1. Demographic associations with childhood overweight, adult obesity and extreme obesity by gender among U.S. adults age 18-64 in 2012-13 (N=28730).

|  | | Sample size  N | Childhood overweight | | Adult Obesity (BMI >30) | | Adult Extreme Obesity (BMI>40) | |
| --- | --- | --- | --- | --- | --- | --- | --- | --- |
|  |  |  | % | OR* (95% CI) | % | OR* (95% CI) | % | OR* (95% CI) |
| **MEN** | | | | | | | | |
| Age | 18-34 | 5152 | 17.2 | 1 | 26.4 | 1 | 3.3 | 1 |
|  | 35-49 | 4240 | 14.0 | **0.82(0.69,0.96)** | 38.7 | **1.90(1.7,2.11)** | 5.5 | **1.82(1.41,2.35)** |
|  | 50-64 | 4001 | 12.3 | **0.67(0.57,0.78)** | 39.6 | **1.97(1.76,2.2)** | 5.2 | **1.61(1.22,2.13)** |
| Region | North | 1864 | 15.5 | 1 | 31.6 | 1 | 4.1 | 1 |
|  | Midwest | 2775 | 15.2 | 0.88(0.7,1.12) | 37.4 | **1.27(1.08,1.49)** | 5.5 | 1.21(0.88,1.68) |
|  | South | 5276 | 14.1 | 0.91(0.75,1.11) | 36.0 | **1.18(1.01,1.37)** | 4.8 | 1.11(0.81,1.53) |
|  | West | 3478 | 14.4 | 0.92(0.74,1.14) | 31.4 | 1.00(0.8,1.24) | 3.8 | 0.99(0.66,1.48) |
| Race/ethnicity | White | 6817 | 15.7 | 1 | 34.4 | 1 | 4.9 | 1 |
|  | Black | 2831 | 12.5 | **0.74(0.62,0.89)** | 38.4 | **1.27(1.12,1.44)** | 5.5 | 1.19(0.93,1.51) |
|  | Amer. Indian | 187 | 18.2 | 1.07(0.70,1.64) | 42.4 | 1.35(0.97,1.87) | 6.9 | 1.39(0.74,2.62) |
|  | Asian | 741 | 10.2 | 0.95(0.70,1.29) | 16.1 | **0.55(0.40,0.77)** | 1.3 | 0.55(0.28,1.08) |
|  | Hispanic | 2817 | 13.7 | 1.14(0.95,1.38) | 37.5 | **1.73(1.49,2.02)** | 3.7 | 1.32(0.92,1.89) |
| Birthplace | U.S. | 10855 | 16.1 | 1 | 36.0 | 1 | 5.2 | 1 |
|  | Other | 2533 | 7.9 | **0.43(0.35,0.52)** | 26.5 | **0.58(0.51,0.66)** | 1.7 | **0.31(0.21,0.46)** |
| **WOMEN** | | | | | | | | |
| Age | 18-34 | 5332 | 18.5 | 1 | 26.2 | 1 | 5.8 | 1 |
|  | 35-49 | 5226 | 14.2 | **0.75(0.65,0.86)** | 36.7 | **1.81(1.62,2.03)** | 8.0 | **1.52(1.25,1.84)** |
|  | 50-64 | 4779 | 16.3 | **0.86(0.75,0.98)** | 39.2 | **2.04(1.81,2.29)** | 8.3 | **1.55(1.29,1.86)** |
| Region | North | 2161 | 17.2 | 1 | 31.9 | 1 | 6.6 | 1 |
|  | Midwest | 3108 | 18.0 | 1.05(0.85,1.28) | 35.8 | 1.14(0.94,1.37) | 8.0 | 1.24(0.94,1.65) |
|  | South | 6294 | 16.3 | 0.97(0.79,1.18) | 37.5 | 1.13(0.94,1.36) | 8.3 | 1.17(0.88,1.54) |
|  | West | 3774 | 14.3 | 0.87(0.7,1.08) | 28.9 | 0.91(0.75,1.11) | 5.7 | 0.95(0.67,1.35) |
| Race/ethnicity | White | 7489 | 17.6 | 1 | 31.9 | 1 | 6.7 | 1 |
|  | Black | 3652 | 15.3 | **0.82(0.7,0.96)** | 51.2 | **2.41(2.11,2.75)** | 13.1 | **2.15(1.78,2.61)** |
|  | Amer. Indian | 246 | 23.3 | 1.45(0.96,2.18) | 45.8 | **1.91(1.4,2.61)** | 13.5 | **2.04(1.31,3.16)** |
|  | Asian | 756 | 6.6 | **0.54(0.37,0.79)** | 9.9 | **0.39(0.28,0.54)** | 1.0 | **0.24(0.10,0.57)** |
|  | Hispanic | 3194 | 15.0 | 1.14(0.97,1.34) | 36.7 | **1.86(1.64,2.12)** | 6.9 | **1.48(1.17,1.88)** |
| Birthplace | U.S. | 12590 | 17.9 | 1 | 36.1 | 1 | 8.1 | 1 |
|  | Other | 2746 | 8.4 | **0.48(0.39,0.59)** | 23.8 | **0.55(0.48,0.63)** | 3.3 | **0.45(0.35,0.58)** |

*ORs are adjusted for demographic variables: age, region, race/ethnicity, and birthplace.

Supplemental Table 2. Childhood psychosocial challenges by gender among U.S. adults age 18-64 in 2012-13 (N=28730).

|  |  | Male (N=13393) | | Female (N=15337) | | p-value |
| --- | --- | --- | --- | --- | --- | --- |
| Poverty | Yes | 2606 | 17.58 | 3454 | 18.68 | **0.024** |
|  | No | 10471 | 82.42 | 11563 | 81.32 |  |
| Maltreatment (Verbal, Physical, or Sexual abuse) | Yes | 3639 | 25.91 | 5003 | 32.56 | **<0.0001** |
|  | No | 9669 | 74.09 | 10164 | 67.44 |  |
| Parental Death | Yes | 1187 | 8.22 | 1384 | 8.35 | 0.11 |
|  | No | 11995 | 91.78 | 13686 | 91.65 |  |
| Parental Separation | Never together | 2165 | 12.71 | 2689 | 13.68 | **0.004** |
|  | Divorced | 2940 | 21.48 | 3578 | 22.32 |  |
|  | Always together | 8275 | 65.81 | 9060 | 64.00 |  |
| Parental mood/anxiety disorder | Yes | 5335 | 41.59 | 7213 | 50.41 | **<0.0001** |
|  | No | 8058 | 58.41 | 8124 | 49.59 |  |
| Parental substance use disorder | Yes | 4408 | 32.21 | 5707 | 36.91 | **<0.0001** |
|  | No | 8985 | 67.79 | 9630 | 63.09 |  |
| Major Depressive Disorder | Yes | 500 | 3.76 | 1026 | 7.39 | **<0.0001** |
|  | No | 12893 | 96.24 | 14311 | 92.61 |  |
| Anxiety disorder | Yes | 753 | 5.9 | 1560 | 11.2 | **<0.0001** |
|  | No | 12640 | 94.1 | 13777 | 88.8 |  |
| PTSD | Yes | 198 | 1.36 | 586 | 3.80 | **<0.0001** |
|  | No | 13195 | 98.64 | 14751 | 96.20 |  |
| Alcohol use disorder | Yes | 519 | 4.18 | 447 | 3.29 | **0.01** |
|  | No | 12874 | 95.82 | 14890 | 96.71 |  |
| Drug use disorder | Yes | 443 | 3.59 | 338 | 2.32 | **<0.0001** |
|  | No | 12950 | 96.41 | 14999 | 97.68 |  |
| Tobacco use Disorder | Yes | 586 | 5.00 | 535 | 3.85 | **0.0005** |
|  | No | 12807 | 95.00 | 12807 | 96.15 |  |
| Childrearing | Yes | 397 | 2.17 | 1674 | 8.23 | **<0.0001** |
|  | No | 12959 | 97.83 | 13644 | 91.77 |  |
| Did not finish High School | Yes | 1912 | 12.42 | 1991 | 10.98 | **0.0005** |
|  | No | 11481 | 87.58 | 13346 | 89.02 |  |

| Supplemental Table 3: Correlation between childhood psychosocial challenges in the U.S. sample who were not overweight in childhood.  Males (n=11,481) in grey lower triangle, Females (n=12,869) in white upper triangle | | | | | | | | | | | | | | |
| --- | --- | --- | --- | --- | --- | --- | --- | --- | --- | --- | --- | --- | --- | --- |
|  | Childhood Adversities | | | | | | Childhood mental disorders | | | | | | Social Challenges | |
|  | Childhood Poverty | Maltreat-ment | Parental Death | Parental Separation | Parental mood or anxiety disorder | Parental substance use disorder | Major Depressive Disorder | Anxiety | PTSD | Alcohol use disorder | Drug use disorder | tobacco use disorder | Childrearing | Did not finish High School |
| Child Poverty | 1.00 | 0.28 | 0.19 | 0.05 | 0.18 | **0.31** | 0.17 | 0.11 | 0.26 | 0.09 | 0.21 | 0.16 | **0.41** | 0.16 |
| Maltreatment | 0.26 | 1.00 | 0.11 | 0.05 | **0.46** | **0.40** | **0.33** | 0.28 | **0.63** | 0.29 | **0.31** | 0.25 | 0.17 | 0.04 |
| Parental Death | 0.19 | 0.08 | 1.00 | 0.04 | 0.01 | 0.08 | 0.07 | 0.04 | 0.10 | 0.11 | 0.03 | 0.02 | 0.14 | 0.15 |
| Parental Separation | 0.01 | 0.00 | 0.02 | 1.00 | 0.13 | 0.13 | 0.06 | 0.06 | 0.03 | 0.12 | 0.11 | 0.10 | 0.05 | 0.09 |
| Parental mood/anxiety disorder | 0.22 | **0.39** | 0.01 | 0.11 | 1.00 | **0.45** | **0.45** | **0.35** | **0.43** | 0.28 | **0.32** | 0.24 | 0.03 | 0.11 |
| Parental substance use disorder | **0.34** | **0.35** | 0.11 | 0.12 | **0.45** | 1.00 | 0.23 | 0.20 | **0.30** | **0.30** | **0.34** | **0.38** | 0.16 | 0.06 |
| Major Depressive Disorder | 0.19 | 0.29 | 0.13 | 0.08 | **0.43** | 0.23 | 1.00 | **0.43** | **0.48** | **0.32** | **0.41** | 0.29 | 0.02 | 0.01 |
| Anxiety | 0.16 | 0.28 | 0.06 | 0.02 | **0.38** | 0.21 | **0.45** | 1.00 | **0.49** | **0.34** | **0.35** | **0.34** | 0.04 | 0.00 |
| PTSD | 0.22 | **0.43** | 0.20 | 0.04 | **0.37** | **0.34** | **0.45** | **0.43** | 1.00 | **0.38** | **0.34** | **0.34** | 0.15 | 0.08 |
| Alcohol use disorder | 0.18 | 0.23 | 0.02 | 0.12 | 0.21 | 0.28 | 0.29 | 0.22 | **0.31** | 1.00 | **0.61** | **0.57** | 0.03 | 0.02 |
| Drug use disorder | 0.21 | 0.25 | 0.07 | 0.11 | 0.29 | **0.32** | **0.34** | **0.30** | **0.33** | **0.62** | 1.00 | **0.58** | 0.08 | 0.03 |
| Tobacco use disorder | 0.19 | 0.22 | 0.01 | 0.05 | 0.19 | 0.28 | 0.23 | 0.25 | 0.21 | **0.56** | **0.55** | 1.00 | 0.15 | 0.12 |
| Childrearing | 0.25 | 0.07 | 0.09 | 0.08 | 0.03 | 0.09 | 0.01 | 0.00 | 0.05 | 0.14 | 0.11 | 0.18 | 1.00 | **0.42** |
| Did not finish High School | 0.18 | 0.01 | 0.08 | 0.07 | 0.10 | 0.08 | 0.01 | 0.01 | 0.06 | 0.14 | 0.09 | 0.14 | **0.32** | 1.00 |
| Note: Correlations are tetrachoric correlations appropriate for dichotomous variables and values greater than 0.30 are in bold to facilitate interpretation of at least moderate effect size associations | | | | | | | | | | | |  |  |  |

Supplemental Table 4. Separate types of childhood maltreatment predicting Obesity and Extreme Obesity by gender among U.S. adults age 18-64 who were **NOT overweight in childhood** (N=24350).

|  | | | Obesity vs No Obestiy | | | | Difference in OR by Gender | | Extreme Obesity vs No Extreme Obesity | | | | | Difference in OR by gender | | |  |
| --- | --- | --- | --- | --- | --- | --- | --- | --- | --- | --- | --- | --- | --- | --- | --- | --- | --- |
|  |  |  | Male | | Female | | p-value interaction | | Male | | Female | | p-value interaction | | |  |  |
|  |  |  | OR* (95% CI) | | OR* (95% CI) | |  | | OR* (95% CI) | | OR* (95% CI) | |  | | |  |  |
| **Types of CHILDHOOD MALTREATMENT^a^** | | | | | | | | | | | | | | | | | |
| Verbal | Yes | **1.17(1.04,1.33)** | | **1.24(1.11,1.40)** | | 0.52 | | 0.86(0.63,1.17) | | **1.43(1.15,1.79)** | | **0.01** | | |  |  |  |
| Physical | Yes | **1.42(1.19,1.69)** | | **1.20(1.03,1.41)** | | 0.17 | | 1.03(0.70,1.53) | | **1.34(1.04,1.73)** | | 0.26 | | |  |  |  |
| Sexual | Yes | 1.14(0.91,1.42) | | **1.20(1.04,1.39)** | | 0.68 | | 1.24(0.77,1.99) | | 1.28(0.98,1.68) | | 0.91 | | |  |  |  |
|  |  |  | |  | |  | |  | |  | |  | | |  |  |  |
| Frequency (max taken across types) | Never | ref | | ref | |  | |  | |  | |  | | |  |  |  |
|  | Almost Never | 1.12(0.96,1.30) | | 0.98(0.86,1.11) | | 0.57 | | 1.09(0.74,1.62) | | 1.00(0.77,1.30) | | 0.10 | | |  |  |  |
|  | Sometimes | **1.30(1.15,1.47)** | | 1.13(0.98,1.30) | | 0.39 | | 0.97(0.67,1.38) | | **1.37(1.06,1.78)** | | 0.43 | | |  |  |  |
|  | Fairly Often | **1.44(1.14,1.83)** | | **1.29(1.07,1.56)** | | 0.75 | | 1.22(0.68,2.19) | | **1.91(1.31,2.76)** | | 0.40 | | |  |  |  |
|  | Very Often | 1.32(0.97,1.79) | | **1.32(1.05,1.65)** | | 0.58 | | 1.09(0.58,2.04) | | **1.57(1.11,2.22)** | | 0.56 | | |  |  |  |

*ORs are adjusted for demographic variables: age, region, race/ethnicity, and birthplace and all other childhood stressors (except other abuse)

^a^Physical abuse was considered positive if the participant had (a) at least “sometimes” been hit hard enough to cause injury or bruises or (b) had “fairly often” or “very often” experienced pushing, grabbing, shoving, slapping, or hitting (2 items). Verbal abuse was considered positive if the participant had at least “sometimes” (a) had a parent or other adult living in their home threaten to hit or throw something at him/her, or (b) felt that the adult acted in a way that made him/her afraid of physical injury (2 items). Sexual abuse was considered positive if the participant reported that he/she had ever experienced any of the following with an adult or other person when he/she didn’t want to or when he/she was too young to know what was happening (a) been touched/fondled in a sexual way, (b) been made to touch/fondle others in a sexual way, or experienced unwanted (c) attempted or (d) completed sexual intercourse (4 items).
